# Analysis of expression of the PD-1/PD-L1 immune checkpoint system and its prognostic impact in gastroenteropancreatic neuroendocrine tumors

**DOI:** 10.1038/s41598-018-36129-1

**Published:** 2018-12-13

**Authors:** Miguel Sampedro-Núñez, Ana Serrano-Somavilla, Magdalena Adrados, José M. Cameselle-Teijeiro, Concepción Blanco-Carrera, José Manuel Cabezas-Agricola, Rebeca Martínez-Hernández, Elena Martín-Pérez, José Luis Muñoz de Nova, José Ángel Díaz, Rogelio García-Centeno, Javier Caneiro-Gómez, Ihab Abdulkader, Roberto González-Amaro, Mónica Marazuela

**Affiliations:** 1 Services of Endocrinology, Immunology and Molecular Biology Unit, Hospital Universitario de la Princesa, Universidad Autónoma de Madrid, Instituto Princesa, 28006 Madrid, Spain; 2 Service of Pathology, Hospital Universitario de la Princesa, Universidad Autónoma de Madrid, 28006 Madrid, Spain; 3 Service of Pathology, Hospital Clinico Universitario, Universidad de Santiago de Compostela, Santiago de Compostela, 15706 Spain; 4 Service of Endocrinology, Hospital Universitario de Alcalá de Henares, Universidad de Alcalá de Henares, 28805 Madrid, Spain; 5 Service of Endocrinology, Hospital Clinico Universitario, Universidad de Santiago de Compostela, Santiago de Compostela, 15706 Spain; 6 Service of Surgery, Hospital Universitario de la Princesa, Universidad Autónoma de Madrid, Instituto Princesa, 28006 Madrid, Spain; 7 Service of Endocrinology, Hospital Clinico San Carlos, Universidad Complutense de Madrid, Madrid, 28040 Spain; 8 Service of Endocrinology, Hospital Universitario Gregorio Marañón, Madrid, 28007 Spain; 9 Department of Immunology, School of Medicine, Universidad Autónoma de San Luis Potosí, 78210 S.L.P. San Luis, Mexico; 10 Research Center of Health Sciences and Biomedicine, Universidad Autónoma de San Luis Potosí, 78210 S.L.P. San Luis, Mexico

## Abstract

The immune checkpoint based therapy targeting the programmed death-1 (PD-1) receptor and its PD-L1 ligand has recently been approved for the therapy of different malignant conditions, but not yet for gastroenteropancreatic neuroendocrine tumors (GEP-NETs). In this context, we evaluated the expression of PD-1 and PD-L1 in GEP-NETs and its potential correlations with clinical outcomes. Expression of PD-1/PD-L1 was analyzed by immunohistochemistry in 116 GEP-NETs and 48 samples of peritumoral tissue. In addition, the expression of these molecules was assessed by flow cytometry in peripheral blood mononuclear cells (PBMC) from patients with GEP-NETs (n = 32) and healthy controls (n = 32) and in intratumoral mononuclear cells (TMCs) (n = 3). Expression of PD-L1 and PD-1 was detected by immunohistochemistry in 6% and 1% of tumor tissue samples, respectively, and in 8% of peritumoral tissue samples, for both markers. We also observed that PD-1 expression by TMCs was associated with metastatic disease at diagnosis, and the levels of circulating PD-1+ PBMCs were associated with progressive disease upon follow-ups. In addition, circulating PD-1+ PBMCs were significantly correlated with PD-L1 expression by tumor cells. Our data suggest that PD-1/PD-L1 is expressed in 1 to 8% of GEP-NETs, and that this feature is significantly associated with disease evolution (p < 0.01).

## Introduction

Different malignant cells may express molecules that are able to induce an immune response, which may be able to eliminate these tumor cells or interfere with tumor progression and metastases. However, malignant cells may also express different molecules that are able to block or interfere with the activation and proliferation of immune cells, avoiding thus the different mechanisms of tumor destruction by the immune response^1^. In this regard, several receptor/ligand systems (immune checkpoints) that are able to downregulate the immune activation have been characterized, including the CTLA-4/CD80, CD86 and PD-1/PD-L1, PD-L2 molecular systems^2^. Thus, it has been widely described that the interaction of the CTLA-4 or PD-1 membrane receptors with their ligands induce different intracellular signal pathways that inhibit the activation of immune cells, mainly T lymphocytes^3^. Accordingly, the blockade of interaction between CTLA-4 and CD80/CD86 or PD-1 with PD-L1/PD-L2 is able to increase the immune response against tumor cells, exerting thus a significant therapeutic effect^4^. However, although the so called immune checkpoint based therapy with PD-1/PD-L1 blocking agents is useful in a significant proportion of patients with different cancer types^5–8^, many of them are refractory to this type of treatment^9^.

PD-1 is a membrane receptor that under steady state conditions is mainly expressed by a small proportion of conventional T cells and by most T regulatory lymphocytes^10^. However, upon activation (through the T cell receptor or different cytokine receptors, mainly those for IL-2, IL-15 and IL-10), most CD4+ and CD8+ conventional T cells show PD-1 expression^11^. Likewise, it has been described that other immune cell types such as monocytes, macrophages, B lymphocytes or antigen presenting dendritic cells (DC’s) are able to express PD-1^11^.

Two PD-1 ligands have been described, PD-L1 (B7-H1, CD274) and PD-L2 (B7-DC, CD273), which show similar effects but different patterns of expression^12,13^. Thus, PD-L1 is a cell membrane anchored molecule that is detected in a significant fraction of unstimulated conventional T cells and most T regulatory (Treg) lymphocytes^10^. In addition, the expression of PD-L1 can be induced in B cells, monocytes and myeloid and plasmacytoid DC’s^14–17^. Furthermore, PD-L1 expression can be detected in other non-immune cells (for example, vascular endothelium), including tumor cells^18^. In contrast, PD-L2 expression is restricted to some immune activated cell types, mainly B lymphocytes, DC’s and monocytes^10^.

The immune regulatory function of PD-1 receptor has been extensively analyzed. Thus, it has been described that upon interaction with any of their ligands, different intracellular signals are generated, which inhibit the activation pathways induced through the TCR and CD28 in T lymphocytes and through other stimulatory receptors in different immune cell types^19^. According to this, upon PD-1 engagement it is inhibited the activation, proliferation and surveillance of conventional T cells^11^. Conversely, PD-1 is involved in the generation and function of Treg lymphocytes^20^. Therefore, the blockade of PD-1 or their ligands results in an increased reactivity of the immune system, *in vitro* and *in vivo*, in animal models and humans^21^. Accordingly, different genetic polymorphisms of the PD-1 gene (PDCD1) have been associated with autoimmune disease, including type-1 diabetes mellitus and systemic lupus erythematosus^22,23^.

Neuroendocrine tumors (NETs) consist of a heterogeneous group of uncommon neoplasms derived from enterochromaffin epithelial cells. These cells have many functional and structural similarities with normal endocrine cells, including production of hormones and peptides like chromogranin A and synaptophysin, which can cause characteristic hormonal syndromes^24^. These neoplasms include the gastroenteropancreatic neuroendocrine tumors (GEP-NETs), which the primary lesions are usually localized in the gastric mucosa, small and large intestine, rectum, or pancreas. GEP-NETs account for approximately 70% of all NETs^25^. Although GEP-NETs are increasingly being diagnosed, a concomitant improvement in outcomes has not been noted. Unlike other malignancies, the natural history of NETs is difficult to predict and most well-differentiated GEP-NETs show an indolent course, even if metastases are present, whereas others may progress rapidly with median survival duration ranging from 5 to 56 months^26^. Although the first therapeutic option for GEP-NETs is the surgical removal of malignant tissue, complete cure is not possible in many cases. Thus, the characterization of biomarkers of prognosis and therapeutic response is an important issue in this condition. Therefore, we decided to assess the expression of the PD-1 receptor and its main ligand (PD-L1) in GEP-NETs as well as to evaluate its possible relationship with clinical outcome. We found that PD-1 expression is detected in a small but significant proportion of GEP-NETs and that the presence of this receptor is significantly associated to increased malignancy. Our data suggest that immune checkpoint based therapy with PD-1 blockade could be useful in significant fraction of patients with GEP-NETs.

## Results

### PD-L1 expression in GEP-NETs

**Figure 1:**
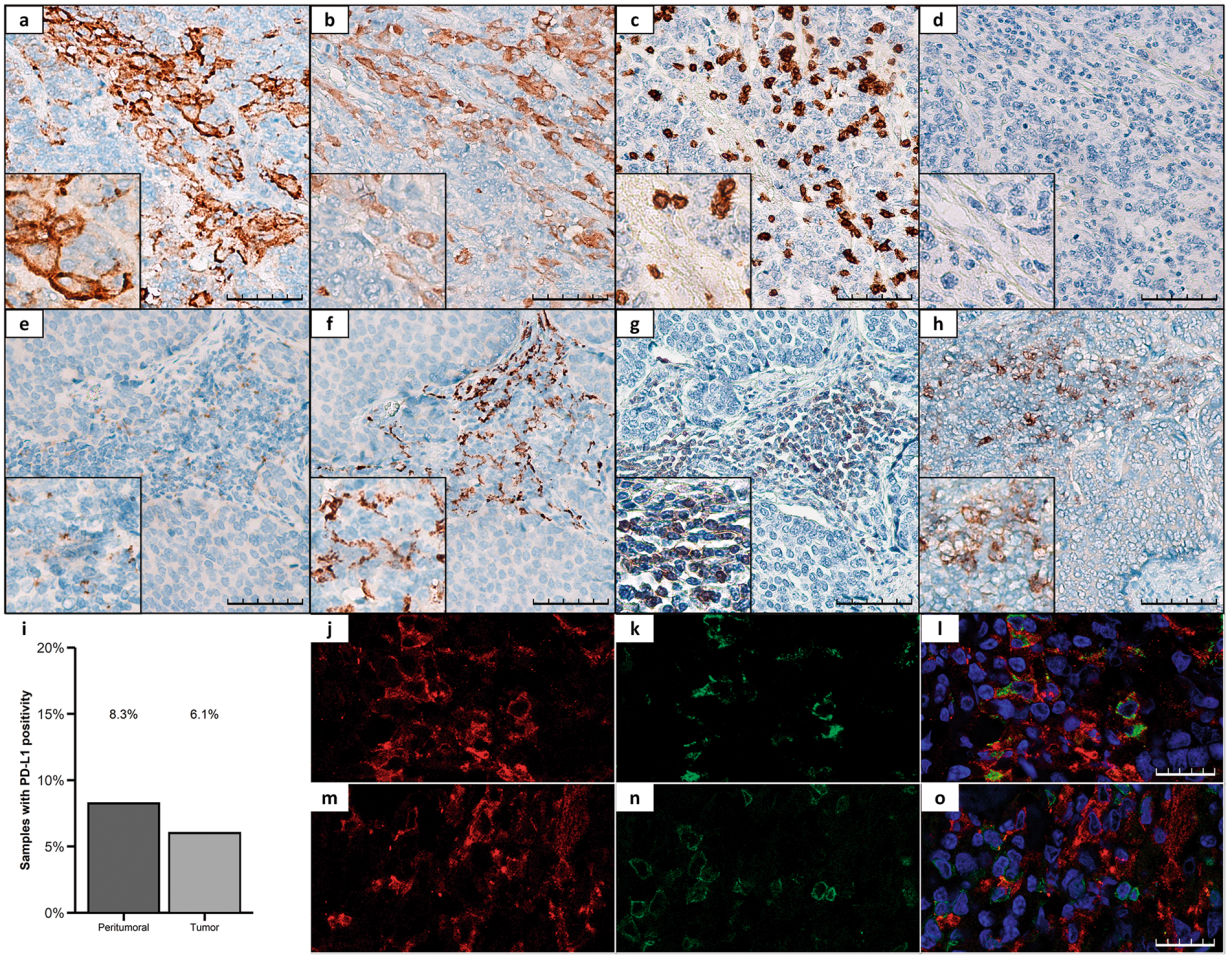
Representative immunohistochemical staining patterns of PD-L1 in GEP-NETs. (**a**–**d**) TMA sections of a G3 pancreatic NET. (**a**) Positive membranous staining for PD-L1 in cancer cells. (**b**) CD163 expression in peritumoral macrophages. (**c**) CD3 expression in peritumoral lymphocytes. (**d**) Negative expression of PD-1. (**e**–**h**) TMA sections of a G2 intestinal NET. (**e**) Positive membranous staining for PD-L1 in peritumoral infiltrating cells. (**f**) CD163 expression in peritumoral macrophages. (**g**) CD3 expression in peritumoral lymphocytes. (**h**) Positive expression of PD-1. Original magnification with 20x and 40x (insets). Scale bar for 100 µm is represented with a line for each panel. (**i**) PD-L1 expression was measured by IHC in a set of TMAs (n = 164) of GEP-NETs. Bar graph values represent the percentage of samples with positivity for PD-L1 expression in the peritumoral and in tumoral tissue. (**j**–**o**) Immunofluorescence (IF) staining in a G3 intestinal NET. (**j**,**m**) Simple IF with PD-L1 (red). (**k**) Simple IF with CD68 (green). (**l**) Double IF with CD68 (green) and PD-L1 (red). (**n**) Simple IF with CD3 (green). (**o**) Double IF with CD3 (green) and PD-L1 (red). Scale bar for 25 µm is represented with a line for each panel.

First, we analyzed the expression of PD-L1 by IHC in tumor and peritumoral tissue samples. As shown in Fig. 1, PD-L1 expression using the clone SP142 was found in 7 of the 116 GEP-NET tumor samples (6.1%) and in 4 of the 48 samples of peritumoral tissue (8.3%). No immunoreaction was detected with the other antibodies (clones 28-8 and 22C3). PD-L1 staining in tumor cells was mainly observed in cell membrane (Fig. 1a). Some infiltrating cells located at the interface between neoplastic cells and stroma showed faint to moderate PD-L1 staining (Fig. 1e). These PD-L1+ cells included CD163+ or CD68+ macrophages (Fig. 1b,f,j–l) but not CD3+ T lymphocytes (Fig. 1c,g,m–o). In some cases, infiltrating cells also showed PD-1 staining (Fig. 1h).

### Characterization of the immune cell infiltrate in GEP-NETs

**Figure 2:**
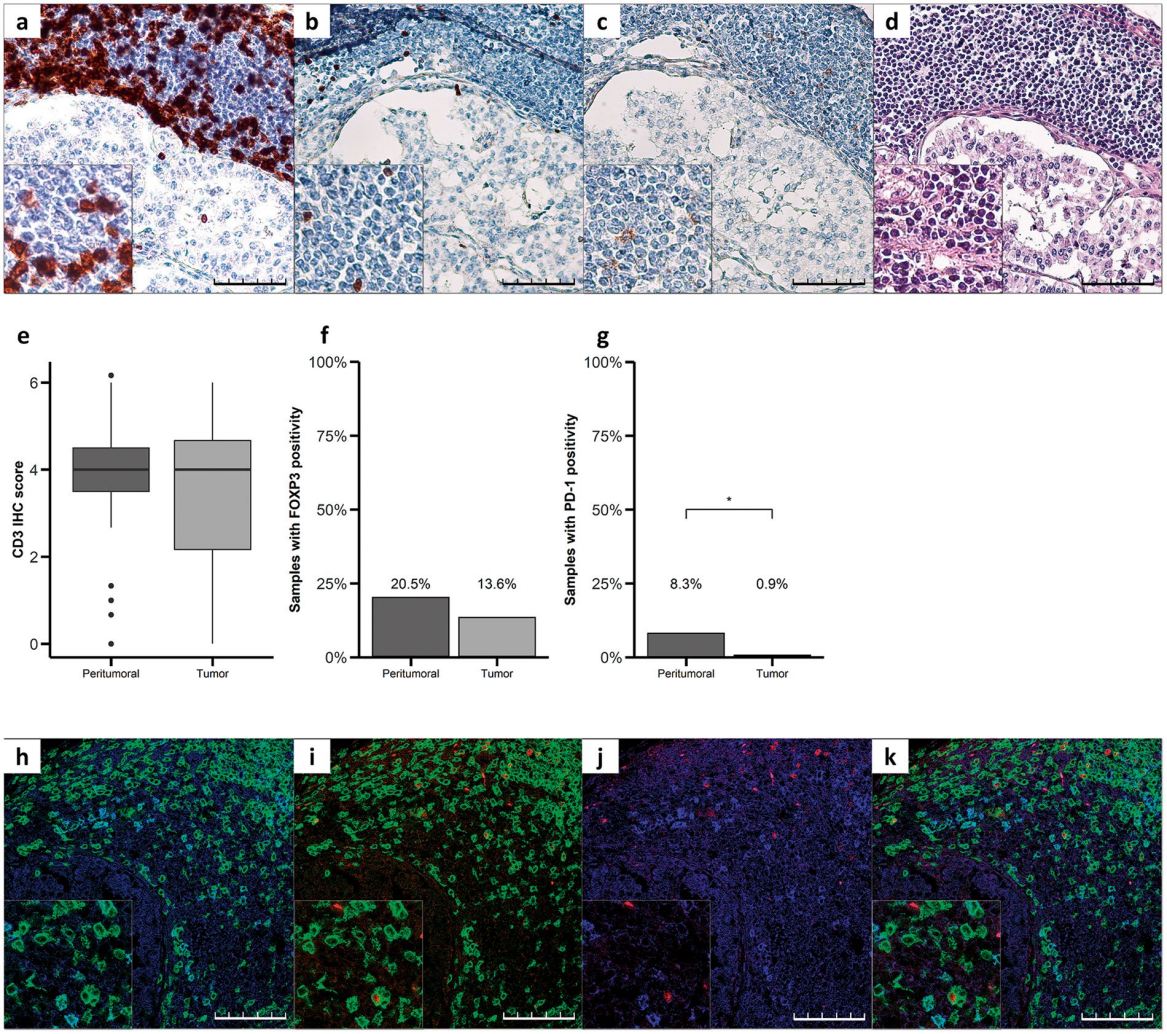
Characterization of the immune cell infiltrate in GEP-NETs. (**a**–**d**) Serial sections of a pancreatic neuroendocrine tumor. (**a**) Intense staining for CD3 in TILs of NET tissue. (**b**) Staining for FOXP3 in some TILs. (**c**) Staining for PD-1 in some TILs, (**d**) hematoxylin-eosin staining. Original magnification with 20x and 40x (insets). Scale bar for 100 µm is represented with a line for each panel. (**e**) CD3, (**f**) FOXP3 and (**g**) PD-1 were measured by IHC in a set of TMAs (n = 164) of GEP-NETs, including primary and metastatic tissue. Values represent boxplot of CD3 IHC score and bar graphs of the percentage of samples with positivity for FOXP3 and PD-1 expression in the tumor tissue and in the peritumoral normal tissue. Asterisks indicate significant differences between tumor and peritumoral tissues (p-values for Fisher’s Exact Test: *p < 0.05). (**h**–**k**) Immunofluorescence showing different markers in TILs: h) Double immunofluorescence with CD3 (green) and PD-1 (blue). (**i**) Double immunofluorescence with CD3 (green) and FOXP3 (red). (**j**) Double immunofluorescence with PD-1 (blue) and FOXP3 (red). (**k**) Triple immunofluorescence with CD3 (green), PD-1 (blue) and FOXP3 (red). Scale bar for 100 µm is represented with a line for each panel.

We then characterized the tumor immune cell infiltrate by using IHC and immunofluorescence. CD3+ T cells were detected in both the peritumoral tissue and between the tumor cells with a CD3 median IHC score of 4 in both groups (Fig. 2a,e; hematoxylin-eosin staining Fig. 2d), whereas FOXP3 expression, a characteristic of Treg cells, was found in 20.5% of peritumoral samples analyzed (Fig. 2b) and in 13.6% of tumor samples (Fig. 2f). There was no significant difference in CD3 or FOXP3 expression score between tumor and peritumoral tissue (p > 0.05). In contrast, PD-1 expression was observed in 8.3% of peritumoral samples and in only 0.9% of tumor tissue (p < 0.05, Fisher exact test, Fig. 2c and g). Furthermore, immunofluorescence microscopy analysis revealed the co-expression of CD3 and PD-1 and CD3 and FOXP3 in the peritumoral tissue (Fig. 2h,i). However, less than 5% cells co-expressed CD3, FOXP3 and PD-1 (Fig. 2j,k).

### Expression of PD-1 is associated to increased malignancy in patients with GEP-NETs

**Figure 3:**
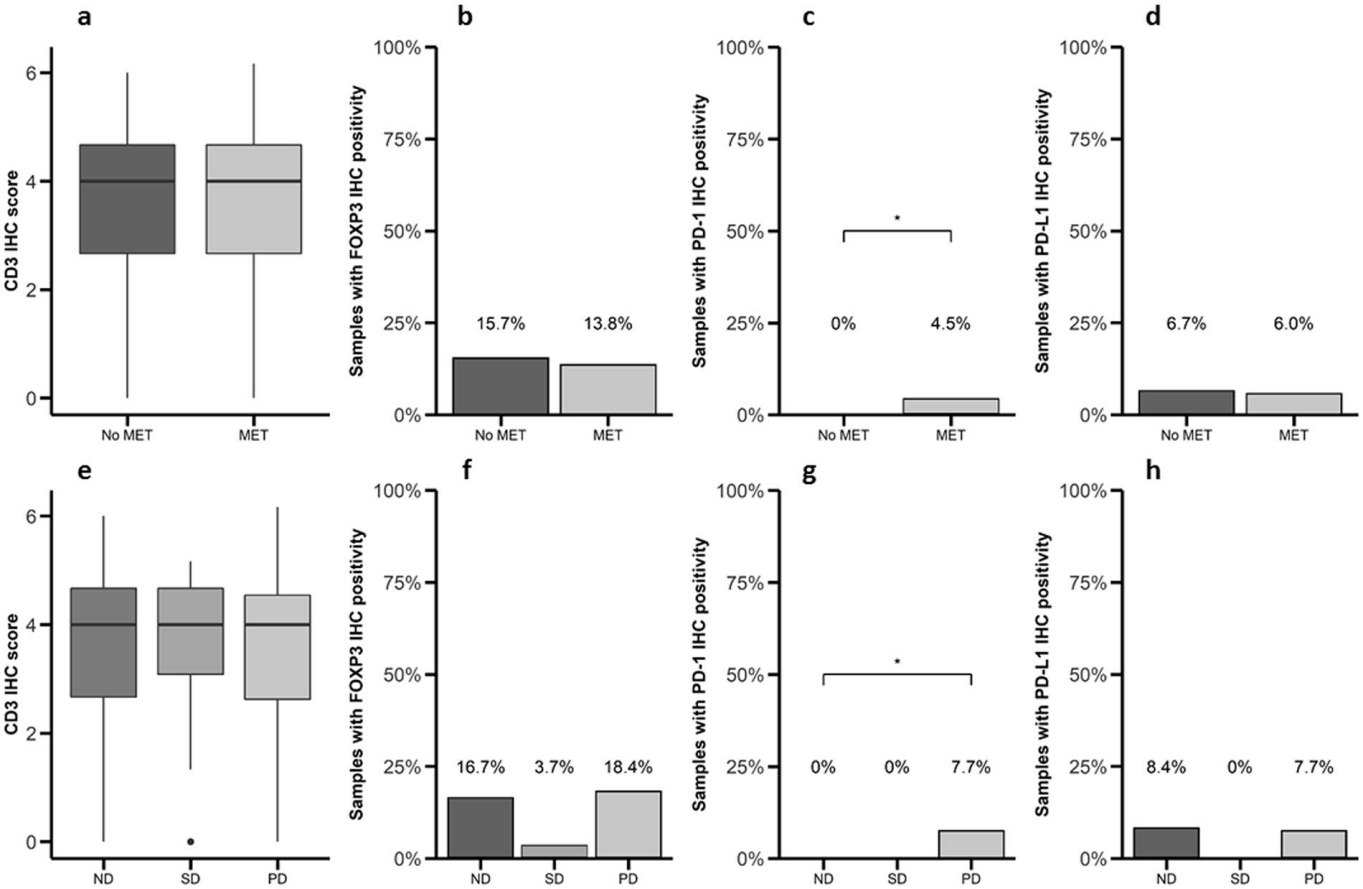
Expression of PD-1 is associated to increased malignancy in patients with GEP-NETs. (**a**,**e**) CD3, (**b**,**f**) FOXP3, (**c**,**g**) PD-1 and (**d**,**h**) PD-L1 were measured by IHC in a set of TMAs (n = 164) of GEP-NETs. Values represent boxplot of CD3 IHC score and bar graphs of the percentage of samples with positivity for PD-1, FOXP3 and PD-L1 expression. (**a**–**d**) Samples were classified based on metastases status at diagnosis: patients with metastasis (MET) or without metastasis (No MET). (**e**–**h**) Samples were classified based on the disease status at follow-up evaluation: (1) non-residual disease, if a complete resection after surgery had been achieved and no tumor relapse/recurrence was evidenced; (2) stable disease, in cases of residual but non-progressive tumor burden; and (3) progressive disease, if tumor growth or new lesions were detected. The median of follow-up was 4.9 years (p25: 2.6–p75: 8.6 years). Asterisks indicate significant differences (p-value for Fisher’s Exact Test: *p < 0.05).

Next, we analyzed the possible relationship between PD-1/PD-L1 expression and malignancy features (Table 1). There was no difference in the immunostaining for CD3 (median IHC score of 4 in both groups, p > 0.05, Fig. 3a), FOXP3 (positive staining in 13.8% and 15.7%, respectively, Fig. 3b) and PD-L1 (positive staining in 6.0% and 6.7%, respectively, Fig. 3d) in tumors from patients with metastatic disease compared to tumors from patients with non-metastatic disease. In contrast, a higher expression of PD-1 was observed in tumors from patients with metastases compared to patients without metastases (4.5 and 0.0%, respectively, p < 0.05, Fig. 3c). A similar trend was observed for CD3, FOXP3, PD-L1 and PD-1 when were compared tumor samples from patients with stable and progressive disease (Fig. 3e–h). However, Kaplan-Meier survival analysis did not show significant differences in the mortality for those patients bearing tumors expressing CD3, FOXP3, PD-1 or PD-L1 (data not shown). These data suggest that PD-1 expression is associated with an increased malignancy in GEP-NETs but not with an increase in mortality rates in this cohort.

**Table 1:** Patients baseline characteristics (n = 110 and sample characteristics (n = 164).

Gender	Number of patients (percentage)
Male	47 (42.7%)
Female	63 (57.3%)
**Age, years**	
<55	46 (41.8%)
≥55	64 (58.2%)
**Stage (ENETS)**	
I	32 (29.1%)
II	20 (18.2%)
III	19 (17.3%)
IV	36 (32.7%)
Unknown	3 (2.7%)
**Disease Type**	
No residual disease	66 (60.0%)
Stable disease	17 (15.5%)
Progressive disease	22 (20.0%)
Unknown	5 (4.6%)
**Primary site**	
Pancreatic NET	48 (43.6%)
Gastrointestinal NET	62 (56.4%)
**Primary tumor size, cm**	
<3.0	69 (62.7%)
≥3.0	37 (33.7%)
Unknown	4 (3.6%)
**Grading (WHO 2010 criteria)**	
G1	66 (60%)
G2	34 (30.9%)
G3	4 (3.6%)
Unknown	6 (5.5%)
**Adjuvant therapy**	
Somatostatin analogues	36 (32.7%)
Interferon	8 (7.3%)
Cytotoxic chemotherapy	5 (4.6%)
Sunitinib	1 (0.9%)
Everolimus	4 (3.6%)
**Sample characteristics (n** = **164)**	
Primary Tumor tissue	104 (63.4%)
Metastatic Tumor Tissue	12 (7.3%)
Non-tumor adjacent tissues	48 (29.3%)

Abbreviations: TNM: Tumor, lymph Node, Metastasis; ENETS: European Neuroendocrine Tumor Society; NET: Neuroendocrine Tumor; WHO: World Health Organization; G1: Grade 1; G2: Grade 2; G3: Grade 3.

### PD-1+ lymphocytes are increased in PBMCs from GEP-NET patients with progressive disease

**Figure 4:**
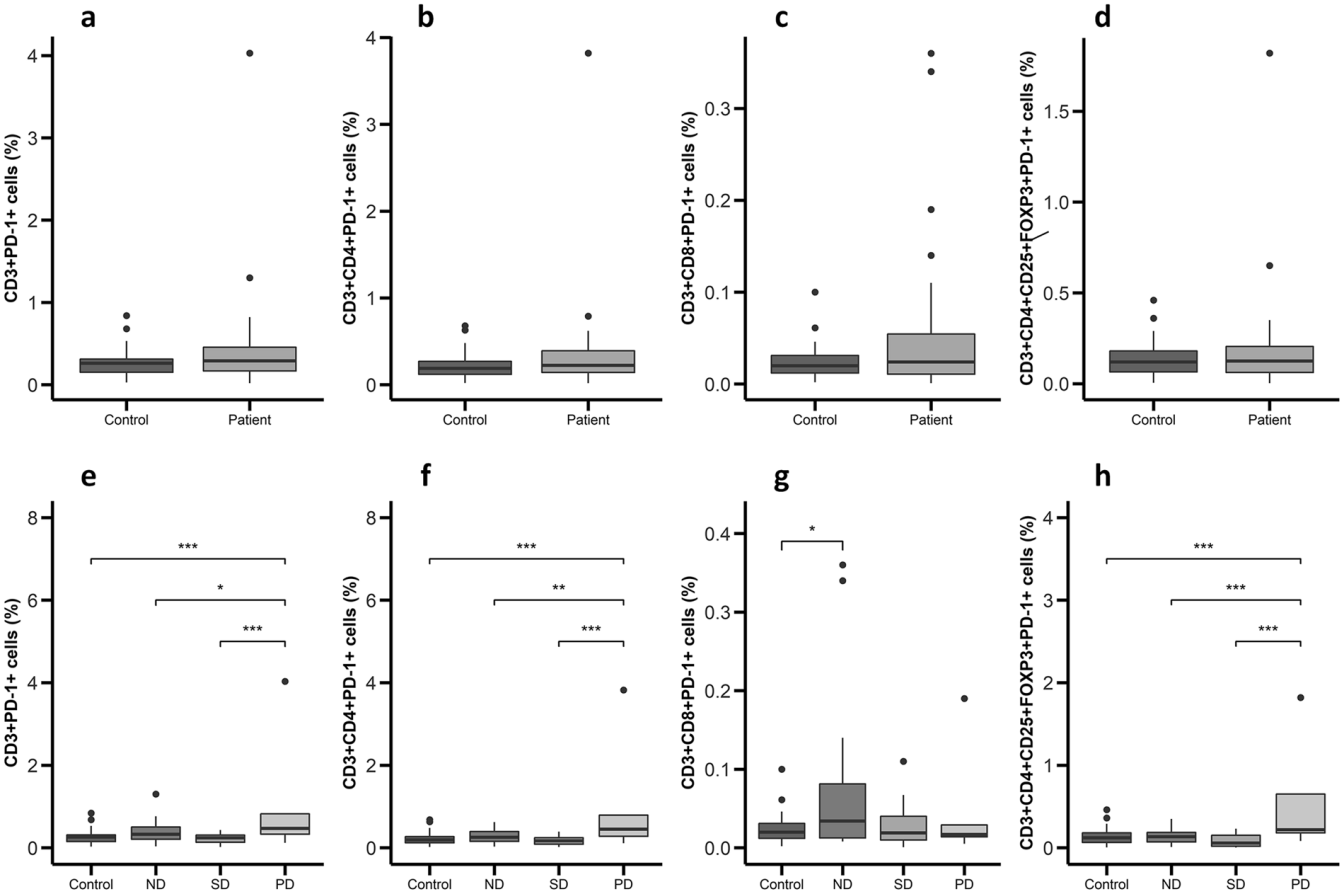
PD-1+ lymphocytes are increased in PBMCs from GEP-NET patients with progressive disease. PBMCs from 32 patients and 32 controls were isolated and incubated with conjugated antibodies directed against CD3, CD4, CD8, CD25, FOXP3 and PD-1. Measurements were made by flow cytometry as stated in ‘Materials and Methods’ and values represent percentage of positive cells for each marker depicted as boxplots. (**a**–**d**) Percentage of PD-1+ cells in healthy controls and patients. (**e**–**h**) Percentage of PD-1+ cells in controls and patients classified according to disease status in non-residual disease (ND), stable disease (SD) or progressive disease (PD). Asterisks indicate significant differences (p-values for Tukey’s test: *p < 0.05, **p < 0.01, ***p < 0.001).

Afterwards, the expression of PD-1 by PBMCs from patients and controls was analyzed. No significant differences were found in the percentages or absolute numbers of CD3 + PD-1+, CD3+ CD4+ PD-1+, CD3+ CD8+ PD-1+ and CD3+ CD4+ CD25+ FOXP3+ PD-1+ cells (Fig. 4a–d and Supplementary Fig. S1) in patients and controls. However, when patients were classified according to the phase of the disease, the levels of CD3 + PD-1+, CD3+ CD4+ PD-1+, and CD3+ CD4+ CD25+ FOXP3+ PD-1+ cells (Fig. 4e,f and h) were significantly higher in patients with progressive disease (0.47%, 0.45% and 0.22%, respectively) compared to those with cured (0.33%, 0.26% and 0.14%, respectively) and stable disease (0.24%, 0.17% and 0.06%, respectively). In contrast, patients with no residual disease had a higher expression of CD8+ PD-1+ PBMCs (0.34%) compared to controls (0.19%, Fig. 4g).

### Simultaneous analysis of peripheral blood and tumor samples

**Figure 5:**
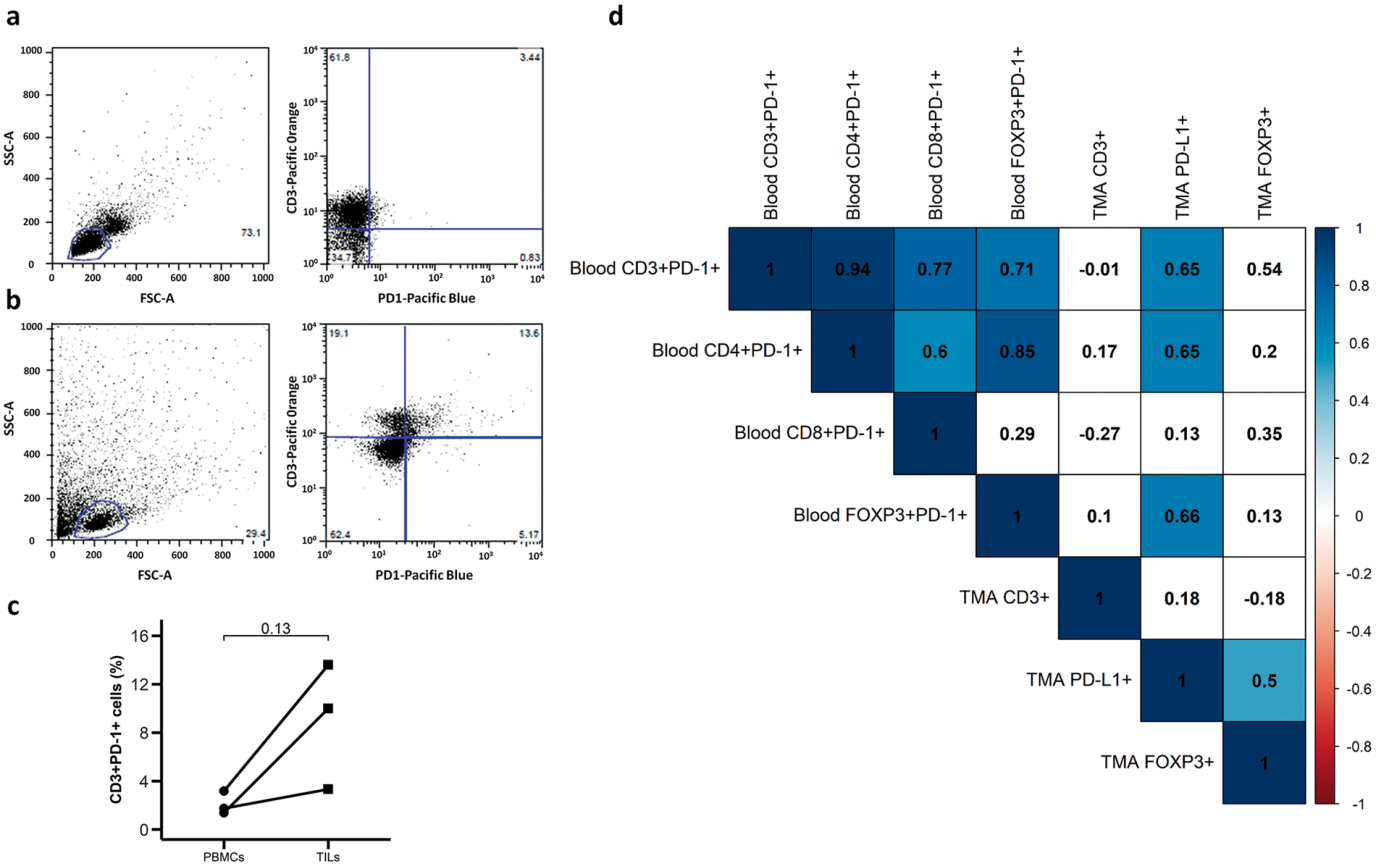
Simultaneous analysis of peripheral blood and tumor samples. Dot plots of CD3+PD-1+ cells from (**a**) peripheral blood and (**b**) tumor specimen from a representative GEP-NET patient. (**c**) Paired analysis of expression of CD3 + PD-1+ cells in PBMCs and TILs from 3 patients with GEP-NETs. Values represent percentage of CD3 + PD-1+ cells in PBMCs and TILs. P-value from paired T test is shown. (**d**) Correlation map for the expression of immune markers in TMAs (IHC) and PBMCs (flow cytometry). Values represent the Spearman’s rank correlation coefficient, rho (ρ). Significant negative correlations are shown in orange and significant positive correlations in blue. Color intensity increases with the magnitude of correlation. White color indicates a non-significant correlation.

Finally, we simultaneously analyzed in three patients the presence of different cell subsets in tumor infiltrating lymphocytes and PBMC samples. As shown in Fig. 5a–c, the proportion of CD3 + PD-1+ cells was higher in mononuclear cells isolated from tumors compared to PBMC (8.97% and 2.09%, respectively). Furthermore, a significant correlation was observed between the percentages of CD3 + PD-1+, CD3+ CD4+ PD-1+ or CD3+ CD4+ CD25+ FOXP3+ PD-1+ lymphocytes in PBMCs and the level of PD-L1 expression detected by IHC in tumor tissue (p < 0.05, Fig. 5d).

## Discussion

GEP-NETs are uncommon neoplasms that are increasingly being diagnosed. The rise in their incidence and the limited available therapeutic options have created an urgent demand for new treatment options. In this regard, cancer immunotherapy is a promising strategy in different malignant tumors. Thus, the immune checkpoint based therapy targeting PD-1 and PD-L1 have recently been approved for their use in patients with different malignant conditions, including melanoma and lung and renal cancer^27^, and it is very feasible that this type of immunotherapy could be useful in other many tumors, including GEP-NETs^28^. Accordingly, we decided to analyze the expression of the PD-1/PD-L1 immune checkpoint in tissue samples and peripheral blood from patients with GEP-NETs.

PD-L1 is expressed by a significant fraction of unstimulated conventional T cells and most Treg lymphocytes^10^. In addition, the expression of PD-L1 can be induced in B cells, monocytes and myeloid and plasmacytoid dendritic cells^14–17^. Furthermore, PD-L1 expression can be detected in other non-immune cells (for example, vascular endothelium), including tumor cells^18^. It has been described that the induction of PD-L1 expression by immune and non-immune cells is mainly mediated by the pro-inflammatory cytokines IFN-γ and TNF-α^29–31^. It has also been stated that PD-L1 expression by tumor cells is the best predictive marker for a good response to PD-1/PD-L1 blockade therapy^32^. In our study, only seven tumor samples (6.1%) and 8.3% of peritumoral tissue showed PD-L1 expression. In this regard, there are several controversial reports on the expression of the PD-1/PD-L1 system in NETs and its possible association with tumor stage and disease outcome^28^. Thus, Fan Y *et al*., have recently reported that the survival time of patients with pulmonary NETs is significantly associated with PD-L1 expression by tumor cells and PD-1 expression by tumor-infiltrating lymphocytes, and that patients with negative PD-1/PD-L1 expression have better prognoses^33^. An additional report^34^ showed PD-L1 expression in only 10% of large cell neuroendocrine carcinoma and 5.8% of small cell lung cell cancer tumors, with no expression in other types of lung neuroendocrine tumors; in this study no significant association was observed between PD-L1 expression and overall survival. Furthermore, Schultheis AM *et al*., have reported a lack of expression of PD-1/PD-L1 by tumor cells in 94 samples of small cell lung carcinomas^35^. However, in this study a significant fraction of tumors (18.5%) showed positive staining for PD-L1 in peritumoral macrophages as well as PD-1 expression by tumor infiltrating T lymphocytes (48% cases); the possible association between PD-L1 expression and disease outcome was not analyzed in this study. Moreover, Kim ST *et al*., detected PD-L1 expression in tumor tissue in 7 out of 32 GEP-NET samples analyzed (21.9%)^36^. In this study, those patients with PD-L1 expression showed a high-grade WHO classification and a diminished survival. Finally, in a very recent report of 62 small intestine NETs, approximately one third of samples showed PD-L1 expression in tumor cells and/or tumor infiltrating lymphocytes TILs, with no apparent association with disease stage or patient outcome^28^. We consider that all these studies strongly indicate that the PD-1/PD-L1 immune checkpoint system is expressed in a significant fraction of NETs, in either tumor or immune infiltrating cells. However, the possible relationship between PD-1/PD-L1 expression and disease outcome seems to remain as an interesting issue to be determined. In this regard, we consider that our data regarding the significant association between malignancy or disease progression and PD-L1 expression are of interest. However, It is important to note that the lack of standardization for PD-L1 IHC in terms of the specificity and reproducibility of the available anti-PD-L1 antibodies, the different staining techniques (manual versus automated), the definition of PD-L1 “positive” tumor (cell surface versus cytoplasmic expression, by tumor cells only or by other cells in the tumor milieu, the threshold of positive cells), the interpretative subjectivity, as well as including patients with different tumor grades− could be the explanation of these discordant results. In our study, results were different when using three different anti-PD-L1 antibodies. We used three different antibodies against PD-L1 but only one of them stained PD-L1 in our tumor samples, probably because they are directed against different immunogenic domains. In any case, most of the GEP-NET patients that we studied did not express PD-L1 either on the tumor cells or peritumoral cells and the low percentage of positive tumors could be related to the reasons described above regarding IHC issues and to the low number of G3 tumors in our cohort^37^.

We also analyzed the possible presence of Treg cells expressing PD-1 in tumor or peritumoral tissue. However, we detected that only very few cells showed the co-expression of CD3, Foxp3 and PD-1. This unexpected finding suggests that, in contrast with other tumor types, Foxp3+ Treg cells do not seem to participate in the induction and maintenance of immune tolerance by tumor cells. However, when our patients were classified according to disease status, we found that the levels of circulating CD3+ CD4+ CD25+ Foxp3+ PD-1+ cells were significantly increased in patients with progressive disease, suggesting a role of Treg lymphocytes in disease outcome. These results indicate that PD-1 expression in PBMCs is associated with a worse clinical outcome in GEP-NET patients, confirming the results that we obtained by IHC and results reported by other authors in non-small cell lung cancer^38^. Thus, we consider that the possible participation of Treg lymphocytes in GEP-NETs remains as an interesting a relevant issue to be elucidated.

Taken together, our results suggest that the PD-1/PD-L1 pathway, especially PD-1 expression, may exert a relevant pathophysiological role in GEP-NETs. Specifically, PD-1 could be involved in tumor progression and worsen prognosis in GEP-NET and its expression could be used to predict a patient’s response to anti-PD-1 therapies. However, our results along with the controversial data reported in previous studies strongly suggest that it would be very convenient to perform a multicentric study to analyze a large number of cases. This type of study may provide very valuable information regarding the role of PD-1/PD-L1 expression in the pathophysiology of NETs as well as the possible involvement of Treg cells and the feasibility of immune checkpoint based therapy in this condition.

## Materials and Methods

### Individuals

A retrospective study was performed, including consecutively patients with gastrointestinal and pancreatic NETs with tumor samples available from 5 reference centers in Spain (Hospital Universitario La Princesa, Hospital Clinico Universitario, Hospital Universitario de Alcalá de Henares, Hospital Clinico San Carlos and Hospital Universitario Gregorio Marañón) between 1995 and 2018. A hundred and ten patients with GEP-NETs were studied (62 with gastrointestinal NETs and 48 with pancreatic NETs) (Table 1). All patients were carefully screened for the presence of other malignancies and/or genetic disorders. One patient was carrier of a MEN1 (Multiple Endocrine Neoplasia Type 1) gene mutation. No other apparent genetic abnormalities were found. Complete work-up including history, physical examination and hormone levels was performed in all cases and interpreted by expert endocrinologists (M.M., C.B.C., J.C.A., J.A.D., R.G.C. and M.S.N.), classifying all patients according to the WHO criteria (tumor site and size, angioinvasion, infiltration level, cell proliferation index, immunohistochemical phenotype, and metastases). According to histopathological findings, GEP-NETs were classified as G1, G2 or G3 (Table 1). All tumors were reviewed (tumor site and size, angioinvasion, infiltration level, cell proliferation index, immunohistochemical phenotype, and metastases) by expert pathologists (J.C.T., J.C.G.; A.I. and M.A), and the neoplasms were classified according to the WHO criteria^24^. GEP-NETs were classified as G1, G2 and G3 (Table 1). Cell proliferation activity was determined by counting the number of Ki-67+ cells. Thirty-two healthy age-matched subjects that had complete hormonal work-up were used as controls for cell isolation and flow cytometry analyses.

Patients were managed following current recommendations and guidelines^39^. Elective surgery was the first option of treatment in all cases and adjuvant therapy with somatostatin analogues was administered if evidence of residual disease was observed (Table 1). According to the last follow-up evaluation (until March 2018) patients were classified into three categories according to their clinical status: (1) non-residual disease, if a complete resection after surgery had been achieved and no tumor relapse/recurrence was evidenced; (2) stable disease, in cases of residual but non-progressive tumor burden; and (3) progressive disease, if tumor growth or new lesions were detected. The median of follow-up was 4.9 years (p25: 2.6–p75: 8.6 years). Five patients did not have enough follow-up data for clinical status classification. Survival at the end of follow-up was 86% (15 patients deceased, no survival data in 3 patients).

This project was approved by the Internal Ethical Review Committee of the Hospital de La Princesa, and written informed consent was obtained from all patients prior to inclusion, in accordance with the Declaration of Helsinki.

### Tissue samples

A total of 164 formalin-fixed paraffin-embedded tissues were evaluated by using a tissue microarray (TMA). Of these, 116 were proper tumor samples with pathological diagnosis and 48 corresponded to peritumoral tissue regions. All samples were taken and managed in accordance with regulations and approval of the local Institutional Review Board.

### Immunohistochemistry (IHC)

Tissue sections were dewaxed, rehydrated and washed in phosphate buffered saline 1x (PBS; Lonza, Verviers, Belgium). Epitope retrieval was performed by treating the slides in a PT Link (Dako, Agilent Technologies, Santa Clara, CA, United States) containing an acid or basic solution (as appropriate), preheated to 97 °C, for 30 minutes. Next, endogenous peroxidase was inhibited with a peroxidase-blocking solution (Dako, Agilent Technologies, Santa Clara, CA, United States) for 5 minutes. Afterwards, sections were immunostained with the following primary antibodies: anti-CD3 (polyclonal, ref.: A0452, Dako, Agilent Technologies, Santa Clara, CA, United States), anti-PD-L1 with three different antibodies (clone SP142, Ventana Medical Systems, Roche, Tucson, Arizona, USA; clone 28-8 and clone 22C3, Dako, Agilent Technologies, Santa Clara, CA, United States), anti-PD-1 (clone NAT105, Ventana Medical Systems, Roche, Tucson, Arizona, USA), anti-FOXP3 (clone 236 A/E7, Abcam, Cambridge, UK) and anti-Ki-67 (clone MIB-1, Dako, Agilent Technologies, Santa Clara, CA, United States). Next, sections were incubated with the proper horseradish peroxidase-conjugated secondary antibodies: goat anti-mouse, goat anti-rabbit or rabbit anti-goat (Ref: P0447, P0448 P0449, respectively, Dako, Agilent Technologies, Santa Clara, CA, United States). Finally, sections were incubated with 3,3′-diaminobenzidine (DAB; Dako, Agilent Technologies, Santa Clara, CA, United States), counterstained with hematoxylin (Sigma-Aldrich, St Louis, MO, USA), dehydrated in alcohol, cleared with xylene and mounted. For each section, the approximate percentage of positive cells (proportion score, PS) and staining intensity (intensity score, IS) determined the CD3 staining score (CD3 IHC score). Five different high-power fields at the hot-spot areas of each slide were observed in a blinded manner. The proportion of stained cells in each field were assessed as follows: 1 for 5% stained cells; 2 for 6–25% stained cells; 3 for 26–50% stained cells; and 4 for >50% stained cells. Intensity of overall staining was graded as follows: 0 for negative staining; 1 for light staining; 2 for moderate staining; and 3 for intense staining. The total staining score (TS) for one field was obtained by adding the score of the proportion of stained cells with the score of the staining intensity (TS = PS + IS). The final TS was the mean of the 5 fields. For the rest of markers in tumor cells and in tumor infiltrating lymphocytes (TILs), sections were scored at 5% intervals, and patients with >5% staining were considered positive for that marker. Tonsil tissue served as positive control for the different antibodies.

### Immunofluorescence

Tissue sections were dewaxed, rehydrated and washed in PBS 1x (Lonza, Verviers, Belgium). Epitope retrieval was performed by treating the slides in a PT Link (Dako, Agilent Technologies, Santa Clara, CA, United States) as described above for IHC. Next, sections were incubated for 1 hour with a blocking solution consisting of 2% (w/v) bovine serum albumin (BSA, Roche, Tucson, Arizona, USA) and 10% (v/v) human serum (Sigma-Aldrich, St Louis, MO, USA) diluted in PBS 1x. Then, sections were incubated at 4 °C overnight with the following primary antibodies: anti-CD3 (Ref: A0452, Dako, Agilent Technologies, Santa Clara, CA, United States), anti-PD-1 (Ref: AF1086, R&D Systems, Minneapolis, MN), anti-FOXP3 (Ref: ab20034, Abcam, Cambridge, UK), anti-CD3 (Ref: M7254, Dako, Agilent Technologies, Santa Clara, CA, United States), anti-CD68 (Ref. M0876, Dako, Agilent Technologies, Santa Clara, CA, United States) and anti-PDL1 (SP263, Cat. 790–4905, Roche, Tucson, Arizona, USA). The next day tissue samples were washed with PBS 1x (3 times, 5 minutes each). Afterwards, tissue sections were incubated for 30 minutes with 4′,6-Diamidino-2-Phenylindole (DAPI, Thermo Fisher Scientific, Waltham, MA, USA) and the appropriate secondary antibodies labeled with a fluorophore: donkey anti-goat Alexa Fluor® 488, donkey anti-rabbit Alexa Fluor® 647, donkey anti-mouse Alexa Fluor® 555, goat anti-mouse Alexa Fluor® 488, and goat anti-rabbit Alexa Fluor® 568 (Cat. A-11055, A-31573, A-31570, A-32723, A-11036 respectively, Invitrogen, Thermo Fisher Scientific, Waltham, MA, USA). Finally, sections were washed with PBS 1x (3 times, 5 minutes each), mounted and analyzed with a Leica TCS-SP5 confocal microscope (Leica Microsystems, Wetzlar, Germany).

### Cell isolation

Peripheral blood samples were obtained from 32 patients and 32 healthy controls. Peripheral blood mononuclear cells (PBMCs) were isolated by Biocoll separating solution (Merck, Berlin, Germany)) density-gradient centrifugation. Cellular viability was assessed by trypan blue dye exclusion and it was always higher than 95%. GEP-NET TILs were isolated from 3 surgical specimens. Briefly, tumor tissue was minced and digested with collagenase (1.0 mg/ml; Roche, Tucson, Arizona, USA) for 1 hour at 37 °C and 5% CO_2_. Then, cells were passed through a steel mesh (BD Biosciences, San Jose, CA, USA) and mononuclear cells were isolated by Biocoll separating solutiondensity-gradient centrifugation. Afterwards, TILs were washed and resuspended in RPMI 1640 with GlutaMAX culture medium (Gibco, Thermo Fisher Scientific, Waltham, MA, USA) supplemented with 10% (v/v) fetal bovine serum (FBS) (Hyclone, Logan, UT, USA), penicillin (50 IU/ml) and streptomycin (50 µg/ml) (Sigma-Aldrich, St Louis, MO, USA).

### Flow cytometry analysis (Fluorescence-activated cell sorting, FACS)

Cell surface expression was assessed on freshly isolated TILs or PBMCs by staining with the following monoclonal antibodies: CD3-Pacific Orange (Clone UCHT1, Cat. 561416, BD Biosciences, San Jose, CA, USA), CD4-APC-Cy7 (Clone OKT4, Cat. 317418, Biolegend, San Diego, CA), CD8-FITC (Clone RPA-T8, Cat. 555366, BD Biosciences, San Jose, CA, USA), CD25-Phycoerythrin (Clone M-A251, Cat. 555432, BD Biosciences, San Jose, CA, USA) and PD-1-Pacific Blue (Clone EH12.2H7, Cat. 329916, Biolegend, San Diego, CA). For detection of intracellular expression of FOXP3, cells were permeabilized and fixed with the ‘FOXP3 Staining Buffer Set’ (BD Biosciences, San Jose, CA, USA) following the manufacturer’s instructions, and then they were incubated with the anti-FOXP3-PE-Cy7 monoclonal antibody (Clone PCH101, Ref: 25-4776-42, BD Biosciences, San Jose, CA, USA). Cell analysis was performed with a FACSCanto flow cytometer (BD Biosciences, San Jose, CA, USA).

### Statistical analysis

Data are shown as the median and interquartile range (boxplots) for quantitative variables and as relative percentages of samples (bar graphs) for qualitative variables included in contingency tables. Fisher’s exact test was used for qualitative variables comparisons. The unpaired two-tailed Student t-test was used to compare two independent groups and the paired Student t-test to analyze two related samples. One-way ANOVA was used to compare more than two groups and post-hoc multiple comparisons were done with Tukey’s test (follow-up losses were not included in clinical status comparison). Furthermore, survival data was analyzed by Kaplan-Meier method. Spearman’s rho analyses were performed to find correlations between blood markers and immune markers studied by IHC. Analyses were performed using Stata v. 12.0 for Windows and R version 3.3.2. Package ggplots2^40^ and corrplot^41^ were used for graphics. For statistical significance, p-values smaller than 0.05 were considered statistically significant.

## Electronic supplementary material

Supplementary Figure S1
