# Supplementary material for: Analysis of expression of the PD-1/PD-L1 immune checkpoint system and its prognostic impact in gastroenteropancreatic neuroendocrine tumors
Source: Sci Rep. 2018 Dec 13;8:17812. doi: 10.1038/s41598-018-36129-1 (PMC6292913; doi:10.1038/s41598-018-36129-1)
Supplement: Supplementary file 1 — Supplementary Figure S1 [file 41598_2018_36129_MOESM1_ESM.pdf]

TITLE:

Analysis of expression of the PD-1/PD-L1 immune checkpoint system and its prognostic impact in gastroenteropancreatic neuroendocrine tumors.

AUTHORS:

Sampedro-Núñez Miguel [1], Serrano-Somavilla Ana[1], Magdalena Adrados [2], Cameselle-Teijeiro José M [3], Blanco-Carrera Concepción [4], Cabezas-Agricola José Manuel [5], Martínez-Hernández Rebeca [1], Martín-Pérez Elena [6], Muñoz de Nova José Luis [6], Díaz José Ángel [7], García-Centeno Rogelio [8], Caneiro-Gómez Javier [3], Abdulkader Ihab [3], González-Amaro Roberto [9,10], Marazuela Mónica [1]

Supplementary Figure S1. T regulatory lymphocytes in PBMCs from GEP-NET patients.

PBMCs from 32 patients and 32 controls were isolated and incubated with conjugated antibodies directed against CD3, CD4, CD8, CD25 and FOXP3. Measurements were made by flow cytometry as stated in 'Materials and Methods' and values represent percentage of positive cells for each marker depicted as boxplots. (a-d) Percentage of total CD3, CD4, CD8 and FOXP3 cells in healthy controls and patients classified according to disease status in non-residual disease (ND), stable disease (SD) or progressive disease (PD).

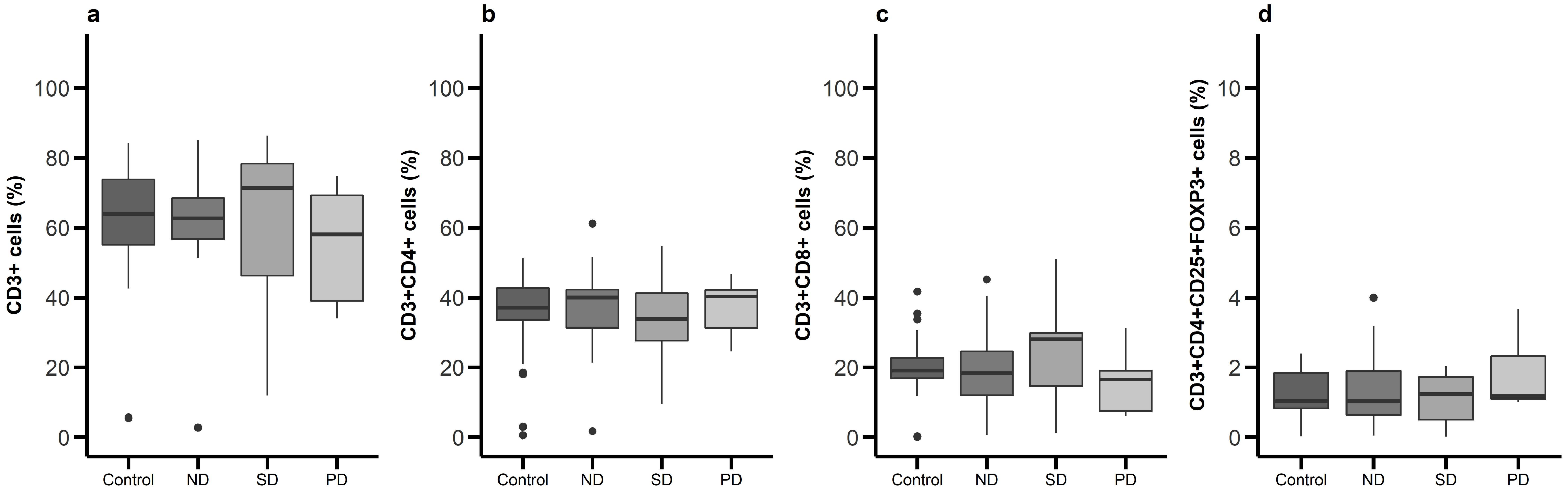
a
b
c
d
CD3+ cells (%)
CD3+CD4+ cells (%)
CD3+CD8+ cells (%)
CD3+CD4+CD25+FOXP3+ cells (%)
0
20
40
60
80
100
0
2
4
6
8
10
Control
ND
SD
PD
